# Tailored Biodegradable and Electroactive Poly(Hydroxybutyrate-Co-Hydroxyvalerate) Based Morphologies for Tissue Engineering Applications

**DOI:** 10.3390/ijms19082149

**Published:** 2018-07-24

**Authors:** Luís Amaro, Daniela M. Correia, Teresa Marques-Almeida, Pedro M. Martins, Leyre Pérez, José L. Vilas, Gabriela Botelho, Senentxu Lanceros-Mendez, Clarisse Ribeiro

**Affiliations:** 1 Center/Department of Physics, Universidade do Minho, 4710-057 Braga, Portugal; luisamaromartins@gmail.com (L.A.); teresaalmeida20@hotmail.com (T.M.-A.); pamartins@fisica.uminho.pt (P.M.M.); cribeiro@fisica.uminho.pt (C.R.); 2 Center/Department of Chemistry, Universidade de Trás-os-Montes e Alto Douro, 5001-801 Vila Real, Portugal; dcorreia@utad.pt; 3 BCMaterials, Basque Center for Materials, Applications and Nanostructures, UPV/EHU Science Park, 48940 Leioa, Spain; leyre.perez@ehu.eus (L.P.); joseluis.vilas@ehu.eus (J.L.V.); 4 Center/Department of Chemistry, Universidade do Minho, 4710-057 Braga, Portugal; gbotelho@quimica.uminho.pt; 5 Macromolecular Chemistry Research Group (labquimac), Department of Physical Chemistry, Faculty of Science and Technology, University of the Basque Country (UPV/EHU), 48940 Leioa, Spain; 6 IKERBASQUE, Basque Foundation for Science, 48013 Bilbao, Spain; 7 CEB—Centre of Biological Engineering, University of Minho, 4710-057 Braga, Portugal

**Keywords:** biomaterials, cobalt ferrites, poly(hydroxybutyrate-co-hydroxyvalerate), tissue engineering

## Abstract

Polymer-based piezoelectric biomaterials have already proven their relevance for tissue engineering applications. Furthermore, the morphology of the scaffolds plays also an important role in cell proliferation and differentiation. The present work reports on poly(hydroxybutyrate-co-hydroxyvalerate) (PHBV), a biocompatible, biodegradable, and piezoelectric biopolymer that has been processed in different morphologies, including films, fibers, microspheres, and 3D scaffolds. The corresponding magnetically active PHBV-based composites were also produced. The effect of the morphology on physico-chemical, thermal, magnetic, and mechanical properties of pristine and composite samples was evaluated, as well as their cytotoxicity. It was observed that the morphology does not strongly affect the properties of the pristine samples but the introduction of cobalt ferrites induces changes in the degree of crystallinity that could affect the applicability of prepared biomaterials. Young’s modulus is dependent of the morphology and also increases with the addition of cobalt ferrites. Both pristine and PHBV/cobalt ferrite composite samples are not cytotoxic, indicating their suitability for tissue engineering applications.

## 1. Introduction

Tissue engineering aims to restore lost or damaged tissues or organs [1]. With this purpose, the most common approach is the use of scaffolds capable of providing structural support for cells to attach and differentiate into specific tissues [1,2]. Following a biomimetic approach, these scaffolds must also provide biophysical stimuli emulating the native properties of the extracellular matrix (ECM) and hence enhancing cell differentiation [1,3].

The design of the scaffold is a complex process since it must accomplish several requirements, namely to be biocompatible to avoid inflammatory responses and biodegradable so it will gradually give place to new tissues [4,5]. The scaffold morphology is an important parameter since it should mimic the structure of the native ECM, promoting differentiation into specific cell lines [1,2]. Thus, it must be tailored according to the application [3]. Tailored mechanical properties are also important for the scaffold does not break during handling [4] and to mimic cell natural environment. Therefore, the choice of the used material is the key factor to success. Some smart materials like poly-l-lactic acid (PLLA) [6], polyvinylidene fluoride (PVDF) [7], polyhydroxybutyrate (PHB) [8], and polyhydroxybutyrate-co-hydroxyvalerate (PHBV) [9] have been successfully used to produce scaffolds. PVDF, PLLA, PHB, and PHBV are piezoelectric, producing local electric potentials upon mechanical stimulation [10,11,12]. Many tissues in the human body show this property, such as skin, bone, muscle, and tendon, thus, smart materials can also provide this stimulus capable of enhancing tissue differentiation [13,14]. The application of smart materials enables the production of such stimuli without the need for wires and power supplies via the mechano-electrical stimuli produced during motion [15].

Among the different electroactive polymers, PHBV is of increasing interest. It is a co-polymer of PHB from the polyhydroxyalkanoates (PHA) family, and shows large potential for tissue engineering, due to its biocompatibility, bioactivity, and piezoelectric properties. It shows better mechanical properties than PHB and is biodegradable [4,16], which represents an advantage with respect to PVDF, the most used piezoelectric polymer for tissue engineering applications in different morphologies [15,17].

In some applications, specific smart materials alone do not have all the needed/desired properties for tissue regeneration, and polymer composites must be developed. In this way, the combination of magnetostrictive nanoparticles and piezoelectric polymers offers the possibility of developing magnetoelectric materials [18], that together with magnetic bioreactors allows the generation of local potentials on the scaffolds, which can be advantageous for mimicking specific environments and stimulate specific tissues regeneration [17,18]. These materials can convert magnetic stimuli into electrical stimuli [19], producing local electric potentials upon magnetic stimulation [20]. Magnetoelectric materials have shown its relevance in neural [21], bone [18,22], and muscle [22] engineering.

In this work, magnetoelectric biodegradable and biocompatible composites are produced. The magnetoelectric composite was developed combining cobalt ferrite particles (CFO) on a PHBV matrix. These particulate composites show important advantages for applications when compared to other magetoelectric composites, such as laminates [18,23] and allow the preparation of porous scaffolds, fibers, films, and spheres. This work demonstrates that both PHBV and the magnetoactive composite PHBV + CFO can be processed in the most common structures for tissue engineering applications without changing the main physico-chemical characteristics of the polymer and maintain its biocompatibility.

## 2. Results and Discussion

### 2.1. Morphological Characterization

**Figure 1:**
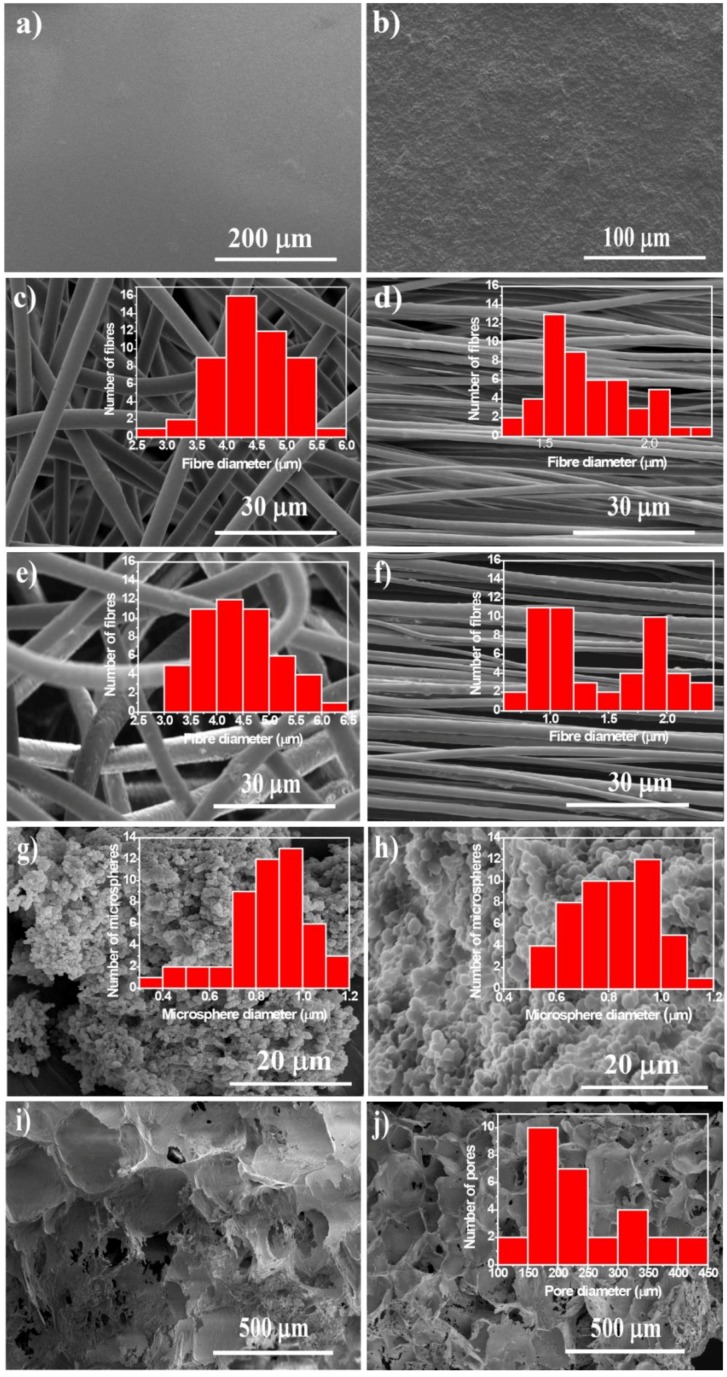
PHBV morphologies: (**a**) neat PHBV films; (**b**) CFO films; (**c**) R fibers; (**d**) O fibers; (**e**) R CFO fibers; (**f**) O CFO fibers; (**g**) neat PHBV microspheres; (**h**) CFO microspheres; (**i**) scaffolds; (**j**) CFO scaffolds. The histograms with the corresponding fiber, sphere, and pore diameters are presented as figure inset.

PHBV and PHBV/CFO composites were processed into different morphologies by different methods in order to obtain a wide range of morphologies suitable for tissue engineering applications. The morphology of the samples, obtained by SEM images, is presented in Figure 1.

Figure 1 shows that both PHBV and PHBV/CFO composites can be successfully processed into different morphologies such as films, random and aligned fibers, microspheres, and scaffolds. The insets show the diameter distribution of fibers, microspheres, and the pore size of the porous scaffolds.

The presence of CFO nanoparticles is observed in the composite films (Figure 1b). The introduction of CFO nanoparticles does not promote changes in the average thickness of the films (≈32 ± 0.9 µm). Random and aligned fibers with the absence of beads and with an average diameter of 4.4 ± 0.6 µm and 1.7 ± 0.2 µm, respectively, were obtained by electrospinning (Figure 1c,d, respectively). Similar to the films, no significant differences were observed in the morphology of the fibers before and after CFO nanoparticle incorporation (Figure 1c–f).

The histograms of Figure 1 show that the highest average fiber diameter is observed for randomly oriented fibers with and without CFO nanoparticles. For the oriented fibers, lower average fiber diameter is observed because the rotating collector promotes the stretching of the as-deposited fibers on the collector, originating thinner fibers. It worth noting that the inclusion of CFO nanoparticles does not promote changes in the surface morphology of the fibers or in their average fiber diameter, with the average diameter of the randomly oriented and oriented fibers being 4.4 ± 0.7 and 1.4 ± 0.5 µm, respectively.

Neat and magnetic microspheres with a smooth surface and an average diameter ranging between 0.8 ± 0.2 µm (Figure 1g,h) were obtained by an oil-in-water emulsion procedure. Again, no significant differences in microsphere diameter were observed when 10% wt of CFO nanoparticles were introduced in the spheres.

Figure 1i,j show that PHBV and PHBV/CFO porous scaffolds can be obtained by the salt leaching method with NaCl crystals as a sacrificial material. A highly porous microstructure is observed with the presence of pores in the same range of the sacrificial material (262–370 µm) [24]. 

### 2.2. Physico-Chemical Properties

To evaluate possible physicochemical modifications in the properties of PHBV after the different processing conditions and the inclusion of the CFO nanoparticles, FTIR-ATR, DSC, and TGA measurements were performed. 

**Figure 2:**
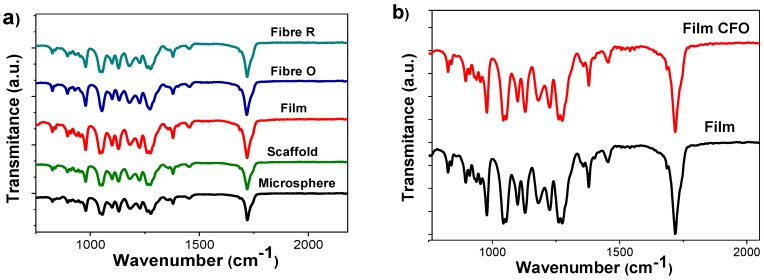
FTIR-ATR spectra of the (**a**) neat PHBV processing into different morphologies and (**b**) PHBV/CFO film composites.

Figure 2a shows the FTIR spectra of neat PHBV processed into the different morphologies. The main characteristic absorption bands of PHBV are observed, namely the absorption bands in the region of 826–979 cm^−1^ and the region of 1227–1478 cm^−1^, which are related to C–H stretching. The absorption bands at 1057, 1133, and 1183 cm^−1^ are assigned to the C–O stretching [25,26] and the absorption band at approximately 1720 cm^−1^ is associated with the C=O vibrational mode [25,26]. No variations are observed, independently of the different processing conditions and morphologies.

The FTIR-ATR measurements were also performed for the different morphologies with CFO nanoparticles to evaluate the influence of the inclusion of the nanoparticles in the chemical structure of PHBV. As a representative example, Figure 2b shows the FTIR spectra obtained for neat and PHBV composite films. It is worth mentoning that no differences were observed in the absorption bands of PHBV after the inclusion of the CFO nanoparticles, indicating that the CFO nanoparticles do not present strong interaction with polymer chemical structure. Similar results were obtained for the fibers, microspheres, and scaffolds.

### 2.3. Thermal Analysis

The thermal characterization of neat PHBV and PHBV/CFO composites was performed by DSC and TGA analysis. 

**Figure 3:**
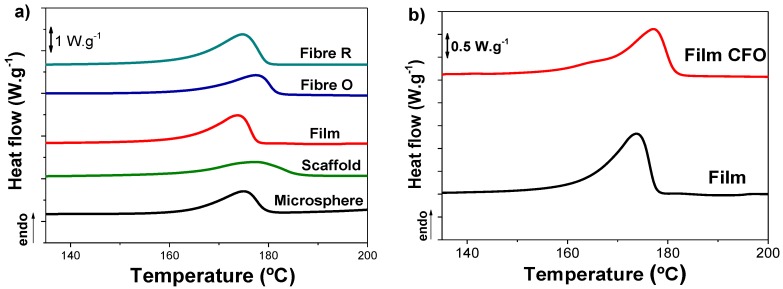
DSC thermograms for (**a**) different neat PHBV morphologies and (**b**) PHBV films and films composites.

Figure 3a shows the DSC thermograms of the different PHBV morphologies and PHBV/CFO composite films. The melting temperature and the enthalpy associated to each endothermic peak is presented in Table 1. All samples exhibit an intense endothermic peak between 160 °C and 180 °C corresponding to the melting peak of PHBV [27]. The inclusion of CFO nanoparticles does not induce relevant modification to this behavior (Figure 3b), the composite films showing a broad peak corresponding to the melting temperature between 160–180 °C. Similar results were obtained for other PHBV composites.

From the enthalpy of the melting peak, the degree of crystallinity (*X_C_*) of neat PHBV morphologies and PHBV composites was obtained by applying Equation (1).

(1) $$X_{c}=\frac{\Delta H_{m}}{\Delta H_{m100}}$$

where Δ*H_m_* is the area of the melting peak and Δ*H_m_*_100_ the enthalpy of 100% PHBV crystalline (146.6 J·mol^−1^) [28]. 

The degree of crystallinity of the different samples is presented in Table 1. All samples show degrees of crystallinity between 40% and 67%, being the largest degree of crystallinity is those of the films and the randomly oriented fibers (above 50%) and lower for the rest of the samples (40% and 50%). These variations in the degree of crystallinity are related with the different crystallizations conditions corresponding to the different processing conditions and morphologies, and have been specifically explored in the literature for related systems [17] for fiber [13,29], sphere [30], scaffold [24,31], and film [32,33] morphologies. The inclusion of CFO nanoparticles induces a decrease of the degree of crystallinity for films and for random fibers, which indicates the CFO nanoparticles act as defects during the crystallization process [34], also hindering spherulite growth [35]. Interestingly, the same behavior is not observed for aligned fibers, where a slight increase is observed (~9%), contrary to the observed in the literature with different polymers [34,36], which can be ascribed to the polymer stretching and acceleration during the jet formation. The increase in the crystallinity degree observed for oriented fibers composites can be attributed to variations in the stretching of the jet during the electrospinning process, due to the modifications of the viscosity and electrical characteristics of the solution.

**Figure 4:**
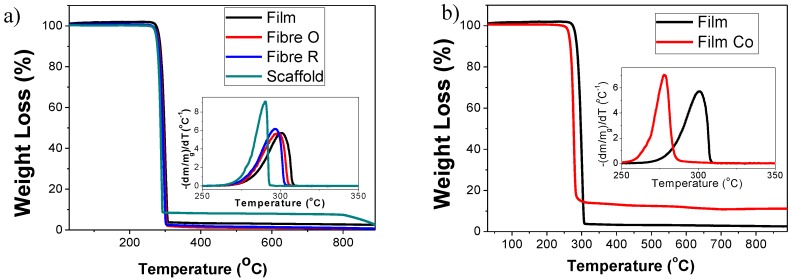
TGA thermograms and corresponding first derivatives for (**a**) different neat PHBV morphologies and (**b**) PHBV films and films composites.

TGA was performed to determine the thermal stability of the different PHBV morphologies and the corresponding composites with 10% wt of CFO. Figure 4 shows the TGA curves of the different PHBV samples as well as the corresponding first derivatives. The different PHBV morphologies without CFO degraded with negligible residue, whereas the composite samples leave a residue of approximately 10% that is related to the CFO content.

Thermal degradation of the different samples occurs in one weight loss step, the onset and peak degradation temperature depend on the processed morphology. Comparing the degradation temperature of the different PHBV morphologies (Figure 4a), it is observed that the scaffold (degradation peak = 290°) is thermally less stable than the rest of the samples, with a degradation peak around 300°. This difference should be attributed to the interaction of the polymer chains with the salt during the preparation process and polymer crystallization, leading to a less stable polymer.

With respect to the polymer composites, the onset and peak degradation temperature of the PHBV films with CFO is shifted towards lower temperatures. The same is observed for the other composites. Thus, the higher thermal conductivity of the nanoparticles with respect to the polymer matrix, lead to an earlier degradation process. Regarding this, contradictory results can be found in the literature. Thus, it has been reported that the introduction of CFO in a PVDF polymer matrix leads to an increase of the thermal stability [37], as well as the introduction of silver nanoparticles in PHBV [38]. Conversely, the introduction of organophilic attapulgite (MAT) in PHBV [39] and magnetite in chitosan derivatives [40] lead to a decrease in the thermal stability of the nanocomposites.

### 2.4. Magnetic Properties of the Composites

**Figure 5:**
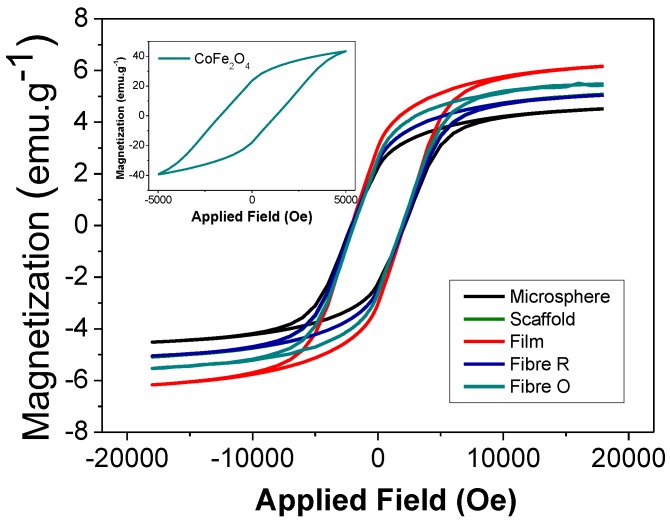
Room temperature hysteresis curves for the CFO/PHBV composites.

The quantification of the magnetic nanoparticle content of the composites was assessed by VSM. Figure 5 shows the magnetization curves of the different PHBV composites determined at room temperature. The inset in Figure 5 represents the magnetization curve of CFO in the form of nanopowder. The CFO nanoparticles reveal a hysteresis loop with coercivity at 24 emu·g^−1^ and a maximum magnetization of 43 emu·g^−1^, at approximately 5000 Oe applied magnetic field [34].

As for CFO nanopowders, the magnetization of the composites increases with increasing magnetic field until saturation, reaching a maximum saturation at approximately 20,000 Oe. By comparing the maximum magnetization saturation of the CFO/PHBV composites, it is observed that it is higher for the PHBV/CFO films (6.2 emu·g^−1^), followed by the scaffolds (Figure 5), which was attributed to different nanofiller content, i.e., some fillers were not integrated in the samples, depending on the processing conditions and sample morphology.

### 2.5. Contact Angle Measurements

Wettability was also assessed for the different samples through sessile drop technique. The results are present in the Table 2.

All samples show contact angles above 90°, presenting an hydrophobic behavior [41]. Fibers show higher contact angles reaching 128 ± 2° while films show the lowest, 90 ± 12°. Differences in wettability between samples are attributed to their morphological differences, which lead to different submicron roughness. For example, the electrospinning technique generates roughness in the sub-micron range since both fibrils and fiber separation are in this order of magnitude [42].

Moreover, the introduction of cobalt ferrite particles leads to an increase in the contact angle of all the morphologies. This increase is associated with the increase of the surface roughness that is related to the introduction of the particles in the polymer [43]. 

### 2.6. Mechanical Properties

**Figure 6:**
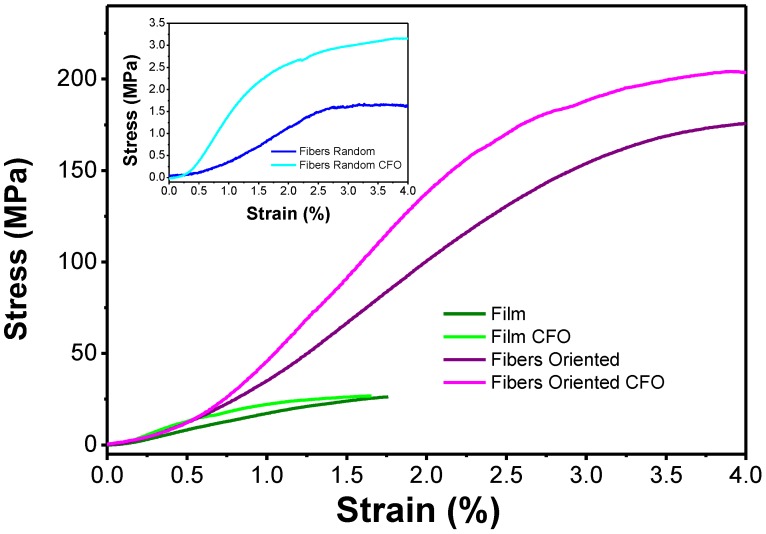
Mechanical stress-strain behavior of the different PHBV samples with and without CFO.

The influence of the morphology on the mechanical response was evaluated by stress-strain mechanical measurements for the films and fibers and compression for the scaffolds (Figure 6). The corresponding Young’s modulus is presented in Table 3.

Comparing the Young’s modulus of the different morphologies, it is verified that the fibers present higher values and the scaffolds the lower. Comparing random and oriented fibers, the lower values of Young’s modulus are explained by the fact that when the fibers are stretched, they are reoriented along the stretching axis with low effective Young’s Modulus, contrary to the oriented fibers that are already aligned along the stretching axis during processing. Therefore, the response of the material and not the reorientation of the matt is measured. Regarding the incorporation of CFO in the polymer matrix, a previous study has shown that the incorporation of CFO in fiber mats increases their Young’s modulus [36] due to the electrostatic interaction between fillers and polymer chains and the proper wetting of the fillers by the polymer. This is in agreement with the presented results, where the introduction of particles leads to an increase of this value in all the morphologies. 

**Figure 7:**
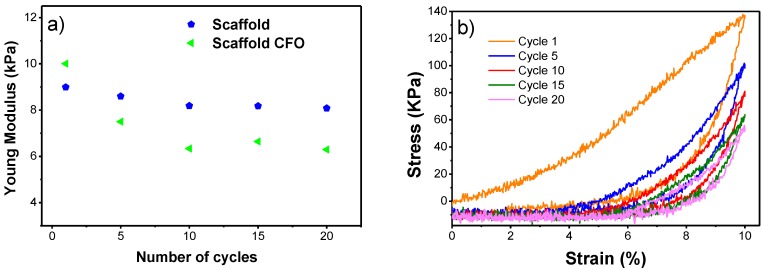
(**a**) Young’s modulus of the PHBV scaffolds with and without CFO along the compression cycles and (**b**) characteristic stress–strain curves of the PHBV scaffolds with CFO for compression assays at 10%.

In relation to the PHBV scaffolds, compression cycles were performed in order to mimic the constant compressions to which the scaffold is subjected during in vivo or in vitro applications. The characteristics stress–strain curve of the PHBV scaffolds with CFO for compression assays at 10% along the different number of cycles is presented in the Figure 7b. The Young’s modulus of the PHBV scaffolds with and without CFO along the compression cycles is presented in Figure 7. It is observed that the Young’s modulus decreases from the first to the second cycle, being more pronounced in the scaffolds with CFO. After that, this value stabilizes and keeps relatively constant up to the 20th cycle. The incorporation of CFO in the PHBV scaffold leads first to an increase of the Young’s modulus value, however, with the increase of the number of compression cycles, this value becomes lower than that of the PHBV scaffold. The addition of CFO leads to some mechanical instability observed by the more pronounced drop in Young’s modulus in the first cycles. Compared with PVDF scaffolds prepared by the same technique, the PHBV scaffolds achieved a slightly smaller Young’s modulus values and both polymers exhibited a drop as cycles progressed [24].

### 2.7. Cytotoxicity Evaluation

**Figure 8:**
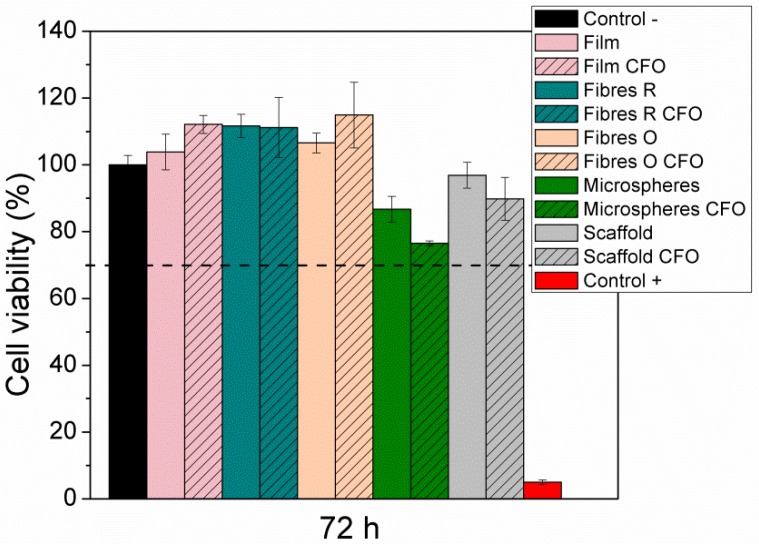
Cytotoxicity assay of MC3T3-E1 pre-osteoblast cells in contact with the as-prepared extraction media exposed to the different PHBV samples for 72 h (relative cell viability was presented as the percentage of the negative control (*n* = 4 ± SD).

The results of the effect of the extract of the different samples on cell viability are presented in Figure 8. It is verified that all the samples do not show any cytotoxic effect (cell viability values higher than 70%). Regarding the samples with CFO in the polymer matrix, it is thus deduced that the particles are efficiently encapsulated, once these particles are cytotoxic [20,21]. In this way, these samples can be used for biomedical applications, and more particularly for tissue engineering applications.

## 3. Materials and Methods

### 3.1. Materials

Poly(hydroxybutyrate-co-hydroxyvalerate), PHBV, (M_w_ = 460.64 g·mol^−1^; HV = 3%, mole fraction), 99% purity, was supplied from Natureplast and polyvinyl alcohol (PVA) (M_w_ = 13–23 g·mol^−1^), 98% purity, from Sigma-Aldrich (Sintra, Portugal). Cobalt ferrite, CFO, nanoparticles with 35–55 nm particle size were purchased from Nanoamor (Katy, TX, USA). Chloroform, 99% purity, and sodium chloride (NaCl), 99% purity, were purchased from Fischer (Porto Salvo, Portugal). All materials were used as received from the provider.

It should be noted that the CFO filler content has been selected as 10% wt in order to provide proper magnetic and magnetoelectric response as in analogous systems [30,44] without hindering the mechanical properties and stability of the structure [36].

### 3.2. Preparation of the Polymer Solution

PHBV was dissolved in chloroform to achieve a polymer concentration of 10% (*w/v*). The solution was prepared under constant magnetic stirring at 40 °C until complete dissolution of the polymer. Magnetic composites were also prepared by the method above described: after the dispersion of 10% (*w/w*) of the CFO nanoparticles in the chloroform solution into an ultrasound bath during 1.5 h, to ensure good dispersion of the CFO nanoparticles and avoid nanoparticles agglomeration, the PHBV optimized concentration of 10% (*w/v*) was added and magnetically stirred to complete polymer dissolution.

These polymer solutions were used to process the PHBV into different morphologies: films, fibers, and scaffolds.

### 3.3. Processing of PHBV into Different Morphologies

#### 3.3.1. Films

**Figure 9:**
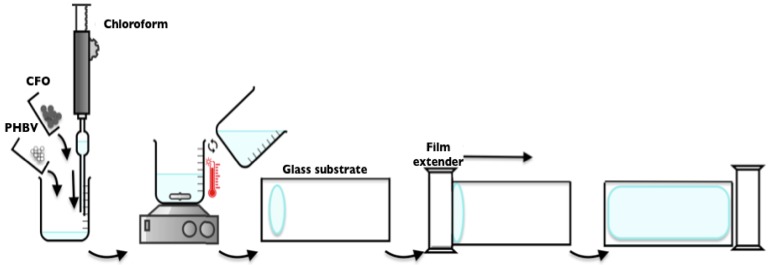
Schematic representation of the processing of the PHBV films by solvent casting.

PHBV and PHBV/CFO composite films were obtained by the solvent casting method. After complete polymer dissolution, the samples were produced by spreading the solution on a clean glass substrate followed by solvent evaporation at room temperature (Figure 9). 

#### 3.3.2. Electrospun Fibers

**Figure 10:**
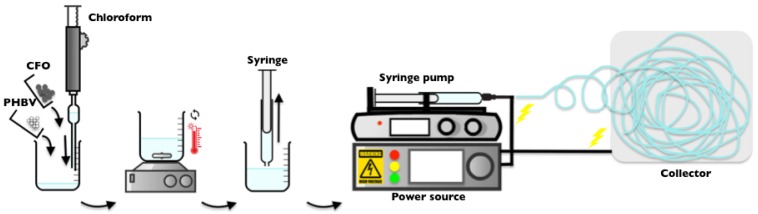
Schematic representation of the processing of the electrospun fibers.

Neat and composite PHBV fibers were obtained by electrospinning (Figure 10). The polymer solution was placed in a plastic syringe (10 mL) fitted with a steel needle with an inner diameter of 0.5 mm. The electrospinning process was conducted by applying a voltage of 20 kV with a PS/FC30P04 power source from Glassman with a solution feed rate of 1 mL·h^−1^. Random and aligned fibers were collected using a grounded collecting plate or a rotating collector, respectively, placed at 15 cm from the needle tip.

#### 3.3.3. Microspheres

**Figure 11:**
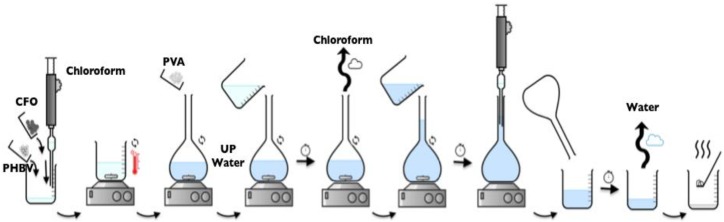
Schematic representation of the oil-in-water emulsion method for the preparation of microspheres.

Neat and magnetic PHBV microspheres were produced, according with the method previously reported, after minor modifications [30] by an oil-in-water emulsion method (Figure 11). After complete polymer dissolution 3% (*w/v*) in chloroform at 40 °C, the mixture was added to 0.5% (*w/v*) of PVA solution in a ratio of 1:10. The emulsified suspension was mechanically stirred at 1000 rpm for 24 h at room temperature, simultaneously with the evaporation of the chloroform. The resulting microspheres were washed with ultra-pure water and air dried at room temperature. 

#### 3.3.4. Scaffolds

**Figure 12:**
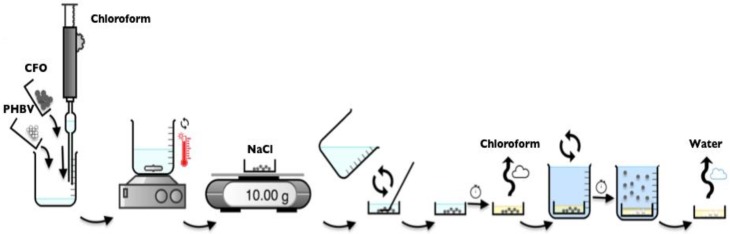
Schematic representation of the preparation procedure of the PHBV scaffolds.

PHBV and PHBV composite scaffolds were produced using the solvent casting via particulate leaching method [2]. The polymer solution was added in a petri dish containing 10 g of NaCl and mixed to obtain a homogeneous dispersion. The solvent was left to evaporate at room temperature. After solvent evaporation, the scaffolds were washed thoroughly with distillate water for three days until complete salt removal. The scaffold was then extracted from the petri dish and dried at room temperature (Figure 12).

Table 4 summarizes the different procedures for the processing of PHBV and PHBV/CFO composites into different morphologies, as well as the corresponding nomenclature. 

### 3.4. Characterization

The morphology of the PHBV and PHBV composites processed by the different methods was analyzed by scanning electron microscopy (SEM) with a FEG-SEM Hitachi with a 3 kV beam acceleration. The analyzed samples were previously coated with a thin gold layer using a sputtering coating (Polaron, model SC502). The size of the prepared microspheres, fibers, and the pore size of the scaffolds were examined by ImageJ software 1.50i.

Infrared measurements (FTIR) were performed in a Jasco FT/IR 4100 (Jasco, Easton, Maryland, USA) apparatus in ATR mode from 4000 to 600 cm^−1^. FTIR spectra were collected after 64 scans with a resolution of 4 cm^−1^. 

Differential scanning calorimetry (DSC) measurements were performed in a Mettler Toledo DSC822e apparatus (Mettler Toledo, Columbus, OH, USA) using a heating rate of 10 °C·min^−1^ under a nitrogen purge (50 mL·min^−1^). The samples were cut into small pieces from the middle region of the films and placed into 40 µL aluminum pans.

Thermogravimetry analyses were performed with a thermal analyzer TGA/SDTA 851e from Mettler Toledo (Mettler Toledo, Columbus, OH, USA). The samples were heated between 25 and 900 °C, at a heating rate of 10 °C·min^−1^ under a nitrogen flow rate of 50 mL·min^−1^.

Mechanical measurements were performed on the different samples in a Shimadzu AG-IS (Shimadzu, Kyoto, Japan) universal testing machine at a test velocity of 1 mm·min^−1^ and room temperature. For the PHBV films and fibers, rectangular samples (25 × 10 mm) with a thickness between 2–30 μm, measured with a digital micrometer Dualscope 603–478 (Fischer, Windsor, CT, USA), were cut and the mechanical measurements were performed in the tensile mode with a loading cell of 50 N. For the PHBV scaffold, cylindrical samples approximately 6 mm diameter and 3.5 mm height were cut and the mechanical measurements were performed in the compression mode with a loading cell of 500 N. The scaffolds were submitted to a compressive-strain cycle load up to 20 cycles at a strain of 10%. The mechanical parameters were calculated from the average of triplicate measurements. The modulus of elasticity (*E*) was determined in the linear zone of elasticity, between 0% and 1% strain, using Hooke’s law, obtaining the effective Young’s modulus of the PHBV samples.

Contact angle measurements were performed at room temperature in a Data Physics OCA20 (Data Physics, Filderstadt, Germany) device using ultra-pure water as drop test liquid. The water drops (3 µL) were deposited on the sample surface and analyzed with SCA20 software provided by the same manufacturer. At least six measurements in each sample were carried out at different sample locations and the average contact angle was taken as the result for each sample.

The magnetic behavior of the composite samples was evaluated at room temperature using a MicroSense EZ7-VSM (MicroSense, Lowell, MA, USA) vibrating sample magnetometer (VSM) from −18,000 Oe to 18,000 Oe.

### 3.5. Cytotoxicity Assay

Indirect cytotoxicity assays were carried out to test if the samples present cytotoxic effect. This assay was adapted from ISO 10993-5 [5] and the cell viability estimated through 3-(4,5-dimethylthiazol-2-yl)-2,5-diphenyltetrazolium bromide (MTT) assay.

Polymer and composite samples were cut into 13 mm diameter discs. These samples were sterilized by exposure to ultraviolet (UV) light for 1 h each side and washed five times in a phosphate buffer saline (PBS) solution for 5 min. After that, the different samples were put into 24-well plates, covered with 500 μL of Dulbecco’s modified Eagle’s medium (DMEM Biochrom, Berlin, Germany) containing 1 g·L^−1^ glucose supplemented with 10% fetal bovine serum (FBS, Biochrom, Berlin, Germany) and 1% penicillin/streptomycin (P/S, Biochrom).

Pre-osteoblastic cells, MC3T3-E1, were seeded at 2 × 10^4^ cells·mL^−1^ (cultured with the same DMEM) on 96-well plates and placed on a 5% CO_2_ controlled atmosphere with 95% humidity at 37 °C for 24 h. Then, the medium was removed from the 96-well plate and replaced by 100 μL of medium previously in contact with the polymer/composite samples. After medium replacement, the 96-well plate was placed for an additional 72 h in standardized culture conditions as mentioned above. A solution of 20% dimethyl sulfoxide (DMSO) was used for positive control. After this incubation time, the cultured medium was again replaced by a 10% MTT solution on DMEM. After incubation for 2 h, MTT crystal was dissolved in DMSO and read at 570 nm on a spectrofluorimeter. Cell viability was calculated according to Equation (2) [5].

(2) $$Cell\;viability\;\left(\%\right)=\frac{Sample\;absorbance}{Positive\;control\;absorbance}\times 100$$

## 4. Conclusions

Different PHBV morphologies—film, fibers, microspheres, and scaffolds—with and without CFO were successfully obtained. The physico-chemical, thermal, magnetic, and mechanical properties of the PHBV pristine and composites samples were evaluated. All the samples produced present a hydrophobic behavior. It is verified that the introduction of cobalt ferrites induces changes in the degree of crystallinity: a decrease in the film (≈8%) and randomly oriented fibers (≈21%) and an increase in the aligned fibers (≈9%). Relatively to the thermal degradation, it was observed that the scaffold is thermally less stable than the others morphologies and that the CFO introduction leads to an increase of the thermal stability. The mechanical properties depend on the morphology (the fibers present higher values and the scaffolds the lower) and the addition of cobalt ferrites improves this value, being more pronounced in the aligned fibers. Their cytotoxic behavior was also evaluated and it was verified that all the produced samples were not cytotoxic, indicating their suitability for tissue engineering applications.

## Figures and Tables

**Table 1:** *T_m_*, Δ*H_m_*, and crystallinity degree of all PHB and PHBV composite samples. The associated error is ±2%.

Sample	*T_m_* (°C)	Δ*H_m_* (J·g^−1^)	*X_c_* (%)
Film	174	82	56
Film/CFO	177	70	48
R fibers	175	98	67
R/CFO fibers	177	67	46
O fibers	177	65	45
O/CFO fibers	179	79	54
Microsphere	175	57	39
Microsphere/CFO	174	57	39
Scaffold	177	63	43
Scaffold/CFO	182	61	42

**Table 2:** Water contact angle measurement for the different samples (mean ± SD).

Film	Film CFO	Fibers O	Fibers O CFO	Fibers R	Fibers R CFO	Scaffold	Scaffold CFO
90 ± 12°	96 ± 4°	103 ± 11°	119 ± 5°	125 ± 2°	128 ± 2°	97 ± 13°	106 ± 9°

**Table 3:** Young’s modulus of the different PHBV samples. Values shown as mean ± SD.

Morphologies	E (MPa)
Film	17 ± 5
Film CFO	27 ± 5
Fibers R	1.1 ± 0.6
Fibers R CFO	1.7 ± 0.5
Fibers O	66 ± 41
Fibers O CFO	83 ± 9
Scaffold	8.9 × 10^−3^ ± 1.7 × 10^−3^
Scaffold CFO	1.3 × 10^−2^ ± 6.4 × 10^−4^

**Table 4:** Procedures for the processing of PHBV and PHBV/CFO composites into different morphologies.

Nomenclature	Composition	Morphology	Processing Technique
Film	PHBV	Film	Solvent-casting
Film CFO	PHBV, CoFe_2_O_4_		
Fibers R	PHBV	Randomly oriented fibers	Electrospinning
Fibers R CFO	PHBV, CoFe_2_O_4_		
Fibers O	PHBV	Oriented fibers	
Fibers O CFO	PHBV, CoFe_2_O_4_		
Microspheres	PHBV	Microspheres	Oil/water emulsion
Microspheres CFO	PHBV, CoFe_2_O_4_		
Scaffold	PHBV	3D Scaffold	Solvent-casting/particulate leaching
Scaffold CFO	PHBV, CoFe_2_O_4_		
